# Supply and Demand-Related Decisive Factors in the Utilization of Non-Medical Community Healthcare Services among Elderly Chinese

**DOI:** 10.3390/ijerph18010228

**Published:** 2020-12-30

**Authors:** Zhao Yu, Lijian Wang, Tolulope Ariyo

**Affiliations:** School of Public Policy and Administration, Xi’an Jiaotong University, No 28 Xianning West Road, Xi’an 710049, Shaanxi, China; yuzhao@stu.xjtu.edu.cn (Z.Y.); ariyotolu@stu.xjtu.edu.cn (T.A.)

**Keywords:** non-medical community healthcare service, healthcare service utilization, supply -related factors, demand-related factors, elderly population, China

## Abstract

There is little research on the utilization of non-medical community healthcare services among the elderly, compared with that of medical community healthcare services. From the perspective of both supply and demand, based on the survey data from Shaanxi province, this study examined supply-related factors (including service supply, service quality, service charge and service accessibility) and demand-related factors (including service need, individual financial status, family care support and knowledge of service) affecting the utilization of non-medical community healthcare services among the elderly in China by using Poisson regression. The findings show that service supply, service quality, service need and knowledge of service are positively associated with the utilization of non-medical community healthcare services among elderly Chinese, but the other factors identified in previous studies are not significant predictors for the utilization of the services among the elderly in the context of China. To our knowledge, this is the first study to examine both supply-related factors and demand-related factors affecting the utilization of non-medical community healthcare services among elderly Chinese.

## 1. Introduction

### 1.1. Background

An ageing population is an inevitable trend at a certain stage of economic and social development and has become a common challenge for the international community [1]. The size of China’s ageing population is enormous and growth is fast. In 2000, China’s population aged 60 and above reached 130 million and accounted for more than 10% of the total population, which signaled that China had stepped into an ageing society. In 2019, China’s population aged 60 and above was close to 254 million and accounted for more than 18% of the total population, meaning that China had become the nation with the biggest ageing population in the world. In 2050, it is estimated that China’s population aged 60 and above will reach 483 million and account for more than 30% of the total population, which suggests that China will enter an advanced stage of ageing [2]. With the continuous increase of the ageing population, the healthcare service needs of the elderly are sharply expanding in China [3]. In this context, to meet the growing healthcare service needs and support the elderly living at home in community the Chinese government has implemented many targeted policies to accelerate the development of community healthcare services for the elderly in recent years.

Community healthcare services include medical and non-medical services, the former mainly for the treatment needs of the individual and the latter mainly for the care needs of the individual [4]. In China, community healthcare services for the elderly also include medical and non-medical services, and the development of the latter lags far behind that of the former [5,6]. Medical community healthcare services for the elderly are provided by community health centers, including a prevention service, diagnosis service, treatment service, rehabilitation service, etc. [7] However, non-medical community healthcare services for the elderly are provided by community healthcare service centers, including a nutrition service, personal care, handling daily affairs, emergency assistance, etc. [8]. Currently, community health centers cover all urban and rural communities in China [9]. As shown in the “2019 Statistical Bulletin of Civil Affairs Development”, community healthcare service centers covered only 17.97% of urban communities and 23.27% of rural communities in China. However, in addition to inadequate supply of non-medical community healthcare services for the elderly, these services have not been utilized fully, leading to unmet need among elderly Chinese and waste of resources [5]. Therefore, it is necessary to examine the decisive factors in the utilization of non-medical community healthcare services among the elderly in China.

### 1.2. Literature Review

#### 1.2.1. Community Healthcare Service Utilization

Regarding the utilization of overall community healthcare services among the elderly, studies have reported that less utilization was associated with older age, male gender, less education, being single, better health, living in rural areas, etc. [10,11]. However, some studies have pointed out that older age was associated with higher healthcare service utilization [12,13]. Researchers also found that inadequate supply [14,15], low quality [16], high charge [8,17] and lack of accessibility [18,19] were discouraging factors for the utilization of healthcare services among the elderly, and low level of service need [20], poor financial status [5,11], good family care support [21] and lack of knowledge of services [18,19] impeded the utilization of healthcare services among the elderly.

#### 1.2.2. Medical Community Healthcare Service Utilization

Regarding the utilization of medical community healthcare services among the elderly, existing research findings showed that there was a significant gender difference in the utilization of medical healthcare services among the elderly, and females utilized more medical healthcare services than males [22,23]. Elders with poorer health status (including high comorbidity [24], disability [25], frailty [26], overweight or obesity [27], and poorer self-perceived health [28,29]) and better financial status [1,30] were more likely to utilize medical healthcare services. The elderly with medical insurance coverage showed increased medical healthcare services utilization [28], those holding a private health plan tended to visit specialists and those using the public system tended to visit a general practitioner [31]. The barriers of service supply impeded the utilization of medical healthcare services [31], and service accessibility was the important factor influencing the utilization of medical healthcare services among the elderly [32].

#### 1.2.3. Non-medical Community Healthcare Service Utilization

In light of the existing literature, the utilization of non-medical community healthcare services among the elderly has received little attention. The functions of non-medical community healthcare services and medical community healthcare services are different, the former mainly for the care needs of the individual and the latter mainly for the treatment needs of the individual [4]. The providers of non-medical community healthcare services and medical community healthcare services are also different, the former usually provided by community healthcare service centers and the latter mainly provided by community health centers in China [7,8]. Naturally, the factors that influence the utilization of these different classes of community healthcare services among the elderly are also likely to differ. Considering that there has been a relative development gap between both kinds of services and that non-medical community healthcare services have been largely underutilized [5,6], we intend to focus solely on the utilization of non-medical community healthcare services among the elderly.

### 1.3. Analysis Framework

Some of the studies on the utilization of community healthcare services among the elderly were conducted by adopting the Andersen behavioral model [19,21,23,33], which has been one of the most extensively used models that identify the decisive factors in healthcare service utilization [23,34]. The Andersen behavioral model divides the factors impacting on the utilization of healthcare service into predisposing factors, enabling factors and need factors [35]. Predisposing factors refer to the factors that predispose individuals to a higher or lower level of service utilization, including age, gender, education and other individual characteristics. Enabling factors facilitate utilization of services, including individual financial status, family resources (such as family care support) and supply-related factors. Need factors are the immediate reasons for services utilization, primarily referring to health status.

However, through several decades of development, the Andersen model is becoming more and more complex and is plagued with a number of theoretical and conceptual inconsistencies [36]. Relevant studies have shown that predisposing factors in the Andersen model were less susceptible to change [35], and better suited to be thought of as control variables in the empirical analysis [37]. Enabling factors were a mixture of supply-related factors and demand-related factors [28,38]. Additionally, it is reasonable for medical healthcare service utilization, and may not be appropriate for non-medical healthcare service utilization, that need factors are measured in terms of health status. Individuals’ needs for medical healthcare services and non-medical healthcare services are very different [4]. Obviously, old people with hypertension would have a need for medical healthcare services but may have no need for non-medical healthcare services [21].

**Figure 1:**
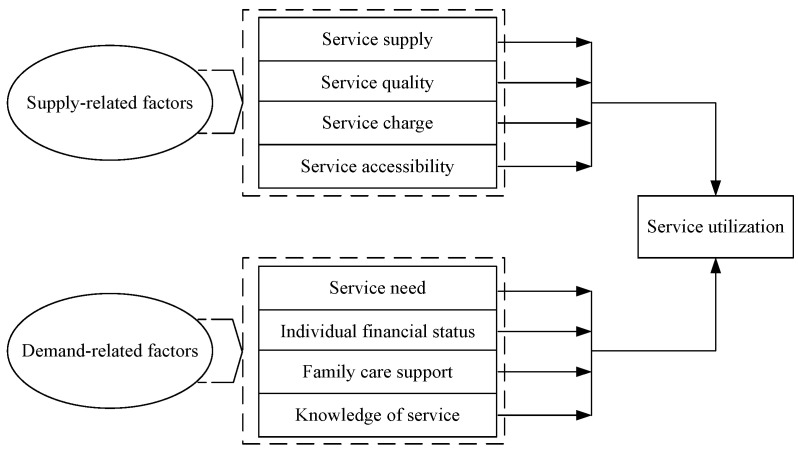
Analytical framework of healthcare service utilization.

Some scholars have pointed out that, as a bridge between the supply and demand of services, healthcare service utilization was largely influenced by supply-related factors and demand-related factors, apart from individual characteristics [14,34]. This provided a more systematic approach to explore the decisive factors in healthcare service utilization [5] and might overcome the shortcomings of the Anderson model. So we adopt this new perspective to reconstruct the Andersen model. First, referring to the research of Lei [37], we consider predisposing factors in the Andersen model as control variables. Second, referring to the research of Zhang and Tong [28], we expand supply-related factors reflected in the enabling factors of the Andersen model into service supply, service quality, service charge and service accessibility. Under the premise of adequate service supply, the general expectations of old people for healthcare services are “high quality, low charge and convenience” [28]. Namely, higher utilization of healthcare services was associated with higher levels of service supply [14,15], service quality [16] and service accessibility [18,19], and lower level of service charge [8,17].Third, referring to the research of Crist et al. [20], we would measure the need factors in the Andersen model in terms of perceived service need instead of health status, and referring to the research of Zhang & Luo [38] we merge service need, individual financial status, family care support, reflected in the enabling factors of the Andersen model, and knowledge of service into demand-related factors. The healthcare service utilization of old people was need-driven, and the transition from service need to effective demand was influenced by individual financial status, family care support and knowledge of service [38]. Namely, higher utilization of healthcare services was associated with higher levels of service need [20] and knowledge of service [18,19], better individual financial status [5,11] and worse family care support [21]. In view of the above, an analytical framework for non-medical community healthcare service utilization among the elderly can be established from the perspective of both supply and demand sides, as shown in Figure 1.

### 1.4. Potential Contribution

Since there is little research on the utilization of non-medical community healthcare services among the elderly, this study focuses solely on non-medical community healthcare services, but not on medical community healthcare services. For the utilization of non-medical community healthcare services among the elderly, we reconstructed the Andersen behavioral model to establish the analytical framework for healthcare service utilization from the perspective of both the supply and demand sides, which may provide a new approach to explore the decisive factors in healthcare service utilization. Based on this analytical framework, we aim to examine the supply and demand-related decisive factors in the utilization of non-medical community healthcare services among the elderly using the survey data from Shaanxi province, China. This may provide important implications for improving the utilization of non-medical community healthcare services among elderly Chinese.

## 2. Materials and Methods

### 2.1. Participant Recruitment

The data used in the article was from the survey conducted in July 2019. The survey team was composed of 24 teachers, doctoral students, and master’s students from Xi’an Jiaotong University. We chose Shaanxi province in China, which is characterized by high ageing and rapid development of non-medical community healthcare services for the elderly. Using multistage stratified random sampling technique, a total of 24 communities (including 12 urban communities and 12 rural communities) were sampled from 12 townships in 6 counties in 3 cities. Taking into account survey costs, we used the non-random sampling technique to choose about 30 old people aged 60 years and above in each community, most of whom lived near a community healthcare service center. To improve the response rate, the investigators were introduced into old people’s houses by community workers and gave each participant a bar of soap and a towel after they had completed the questionnaire. To ensure that all questions in the questionnaire were answered as completely as possible, the investigators directly asked old people questions and filled in the questionnaire. A few old people refused to be investigated. In the end, the team obtained a total of 694 questionnaires.

It should be noted that out of 24 surveyed communities, 8 communities were not covered by community healthcare service centers, including 5 urban communities and 3 rural communities. Therefore, 221 old people from these communities did not answer the questions in relation to the utilization of non-medical community healthcare services, and the corresponding data were excluded. For various reasons, 15 old people from other communities did not complete the questionnaire, and the corresponding samples with missing data also were excluded. Finally, the remaining 458 questionnaires were included in our analysis. Data collection was approved by Xi’an Jiaotong University Ethics Committee. Each participant was voluntary and was informed of the purpose of the survey, and their privacy would be strictly protected.

### 2.2. Variables and Their Measurements

#### 2.2.1. Healthcare Service Utilization

For the purpose of this study, non-medical community healthcare service utilization was defined as a dependent variable in our analysis. Referring to the research of Du and Wang [5], non-medical community healthcare service utilization was measured by four questions asking the respondents whether they had used a nutrition service, personal care, handling daily affairs and emergency assistance in the previous six months, all of which were the most common services provided by community healthcare service centers [6]. For each of the questions, the answers include “yes (1)” and “no (0)”. In the end, the scores were summed to form a total score range between 0 and 4, with higher scores representing higher level of non-medical community healthcare service utilization.

#### 2.2.2. Supply-related Factors

According to the analysis framework in Figure 1, independent variables in this study were categorized as supply-related factors and demand-related factors, and supply-related factors included service supply, service quality, service charge and service accessibility. Similarly to the measurement of non-medical community healthcare service utilization, service supply was measured by four questions asking the respondents whether they knew of nutrition service, personal care, handling daily affairs and emergency assistance offered by community healthcare service centers. For each of the questions, the answers included “yes (1)” and “no (0)”, and the scores were summed to form a total score range between 0 and 4, with higher scores representing higher level of service supply. If the respondents answered that they did not know any of the services offered by community healthcare service centers, they were excluded from the further questions with regard to service quality and service charge. Service quality was measured primarily from the consumers’ perspective [39], and consumers’ satisfaction is the most important parameter for the assessment of service quality [40,41]. So, respondents’ satisfaction with non-medical community healthcare services was used as a proxy for service quality, and the answers included “very dissatisfied (1)”, “dissatisfied (2)”, “average (3)”, ”satisfied (4)”, ”very satisfied (5)”. A higher score represented a higher level of service quality. Referring to the research of Li & Wang [8], service charge was measured by asking the respondents to judge the price of non-medical community healthcare services, with answers ”very low (1)”, “low (2)”, “average (3)”, “high (4)”, “very high (5)”, and a higher score representing a higher level of service charge. Referring to the research of Di et al. [3], service accessibility was measured by asking the respondents to judge the convenience of the community healthcare service center, with answers of ” very inconvenient (1)”, “inconvenient (2)”, “average (3)”, “convenient (4)”, “very convenient (5)”, a higher score representing a higher level of service accessibility.

#### 2.2.3. Demand-related Factors

According to the analysis framework in Figure 1, demand-related factors included service need, individual financial status, family care support and knowledge of service. Similarly to the measurement of non-medical community healthcare service utilization, service need was measured by four questions asking the respondents whether they might need nutrition service, personal care, handling daily affairs and emergency assistance offered by community healthcare service centers in daily life. For each of the questions, the answers included “yes (1)” and “no (0)”, and the scores were summed to form a total score range between 0 and 4, with higher scores representing higher level of service need. Referring to the research of Guo et al. [11], personal annual income was defined as a proxy of individual financial status and was obtained from the sum of various incomes of each old person in 2018. Referring to the research of Peng et al. [21], family care support was measured by asking the respondents whether they received such support in their daily life, with answers of “yes (1)” and “no (0)”. Referring to the research of Chen & Chen [19], knowledge of service was measured by asking the respondents how well they knew non-medical community healthcare services, with answers of ” very little (1)”, “little (2)”, “average (3)”, “well (4)”, “very well (5)”, a higher score representing a higher level of knowledge of service.

#### 2.2.4. Control Variables

Referring to relevant research, we regarded individual characteristics including age, gender, education, marital status, health status, and residency location as control variables. Referring to the research of Fu et al. [1], age referred to the chronological age of the respondents. Referring to the research of Shim et al. [23], gender was grouped as female (0) and male (1). Referring to the research of Di et al. [3], education was grouped as primary school and below (1), middle school (2) and high school and above (3). Referring to the research of Lai [12], marital status was grouped as married (1) and single (0), which included those who reported to be divorced, widowed, and never married. Referring to the research of Zhang and Tong [28], health status was measured by asking the respondents how they felt in terms of health, with answers of ”very poor (1)”, “poor (2)”, “average (3)”, “good (4)”, “very good (5)”. Referring to the research of Di et al. [3], residency location was grouped as urban (1) and rural (0).

In summary, the measurements of variables are shown in Table 1, and the sources of variables are listed in the last column of Table 1.

### 2.3. Statistical Analysis

SPSS version 20.0 (IBM Corp., Armonk, NY, USA) was used in data analysis. First, frequency distribution was used to report individual characteristics of respondents. In view of this, we evaluated the representativeness of the resulting sample. Second, means and frequency distribution were used to report dependent variables and independent variables. Third, we examined the effect of supply-related factors and demand-related factors on non-medical community healthcare service utilization controlling for individual characteristics using Poisson regression. The dependent variable “service utilization” is a typical non-negative integer count data with finite value. As presented in Table 2, the examination of service utilization data showed that the skewness (=1.83) was obviously greater than 1, indicating that the data showed skewed distribution, and the mean (=0.36) was close to the variance (=0.34), suggesting equi-dispersion of the data, approximately. Therefore, Poisson regression was used to address skewed distribution and equi-dispersion of the count data [42]. Additionally, the results of Poisson regression were also basically consistent with those of negative binomial regression, indicating that our choice of Poisson regression is appropriate. We fitted four hierarchical models. The first part incorporated control variables into Model 1. The second part incorporated control variables and supply-related factors into Model 2. The third part incorporated control variables and demand-related factors into Model 3. Lastly, the fourth part incorporated the control variables, supply-related factors, and demand-related factors into Model 4.

## 3. Results

### 3.1. Descriptive Statistics

Descriptive statistics of individual characteristics are shown in Table 3. The rate of the respondents aged 60 to 69 was 51.09%. The majority of the respondents were female (61.14%) and married (72.71%). Approximately half of the respondents (48.91%) had primary school and below education. About 79.0% of the respondents rated their health status average or above. 41.48% of the respondents live in the urban areas, and 58.52% of them live in the rural areas. In the resulting sample, the rate of old women was greater than that of old men, and elderly living in urban areas were fewer than those living in the rural areas. In China, however, the rate of old women was close to that of old men, and the elderly living in urban areas were more than those living in rural areas [43]. Therefore, on account of nonrandom sampling of the elderly, we must admit that the representativeness of the sample was not particularly good.

Descriptive statistics of dependent variable and independent variables are presented in Table 4. Regarding the dependent variable, the mean score of service utilization is 0.36 (SD = 0.58), indicating that the level of utilization of non-medical community healthcare services among the elderly is very low. The data show that the elderly rarely makes use of non-medical community healthcare services, and 67.69% of the elderly have never used any part of the services.

Regarding supply-related factors in independent variables, the mean score of service supply is 1.64 (SD = 1.20), indicating that the level of supply of non-medical community healthcare services is low. Most community healthcare service centers provide only one (38.21%) or two (22.27%) of the services, and therefore non-medical community healthcare services need to be enriched. The mean score of service quality is 3.27 (SD = 1.06), indicating that the quality of non-medical community healthcare services for the elderly is generally average and non-medical community healthcare services are still not very professional. The mean score for service charge is 1.47 (SD = 0.87), indicating that the charge of non-medical community healthcare services to the elderly is generally low. The mean score of service accessibility is 4.21(SD = 1.02), but on account of nonrandom sampling of the elderly, this does not suggest that the accessibility of non-medical community healthcare services is good for all older people, and only indicates that the accessibility of non-medical community healthcare services is good for the respondents.

Regarding demand-related factors in independent variables, the mean score for service need is 1.51 (SD = 1.60), indicating that the elderly’s need level for non-medical community healthcare services is generally low. Nearly half of the elderly (44.54%) say that they don’t need any services offered by community healthcare service centers. The mean for individual financial status is 2.13 (SD = 2.56), indicating that annual income per capita of the elderly reaches 2.13 ten thousand RMB. Of the elderly, 82.53% can receive care support from family members in their daily life, indicating that family care support for the elderly still plays a major role in China. The mean score for knowledge of service is 3.32 (SD = 1.25), indicating that the elderly have an average knowledge of non-medical community healthcare services.

Compared with the mean score for service need (Mean = 1.51), the mean score for service supply (Mean = 1.64) is slightly larger. In this study, however, the measurement of service supply and need are not particularly accurate, and only approximately reflect their respective levels. So we can only judge that the levels of both service supply and need are low. Nevertheless, the mean score for service utilization (mean = 0.36) is obviously smaller than that for service supply or service need. It may be judged that the level of service utilization is lower than that of service supply or service need, indicating that there is an imbalance between supply and demand sides.

### 3.2. Poisson Regression Analysis

Table 5 presents the results from Poisson regression models predicting the level of the utilization of non-medical community healthcare services among the elderly. The −2log likelihood is significant in Model 1 (*p* < 0.001), Model 2 (*p* < 0.001), Model 3 (*p* < 0.001) and Model 4 (*p* < 0.001), indicating that all the four models have significant explanatory power. Furthermore, the change of −2Log likelihood in Model 2 is significant (*p* < 0.001), indicating that there is at least one factor significantly associated with service utilization among supply-related factors. The change of −2Log likelihood in Model 3 is also significant (*p* < 0.001), indicating that there is at least one factor significantly associated with service utilization among demand-related factors. Model 4 is the final model.

**Figure 2:**
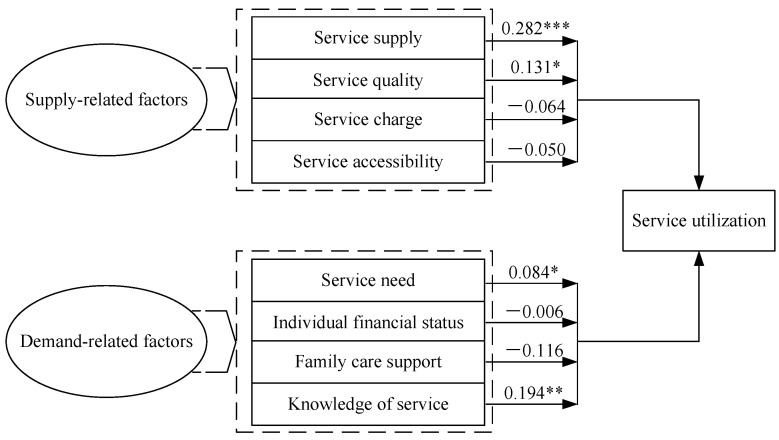
Path of regression model and results of parameter estimation. Note: * *p* < 0.05, ** *p* < 0.01, *** *p* < 0.001.

According to the final model, the results are presented more clearly in Figure 2.

Among supply-related factors, the findings indicate that service supply (B = 0.282, *p* < 0.001) and service quality (B = 0.131, *p* < 0.01) are positively associated with the utilization of non-medical community healthcare services among the elderly. If the other conditions remain unchanged, an increase of 1 unit in service supply results in an increase of 0.325 (=1.325 − 1) unit in service utilization, and an increase of 1 unit in service quality results in an increase of 0.140 (=1.140 − 1) unit in service utilization. However, service charge and service accessibility are not significant predictors for the utilization of non-medical community healthcare services among the elderly.

Among demand-related factors, the findings show that service need (B = 0.084, *p* < 0.01) and knowledge of service (B = 0.194, *p* < 0.05) are positively associated with the utilization of non-medical community healthcare services among the elderly. With the other conditions unchanged, an increase of 1 unit in service need results in an increase of 0.088 (=1.088 − 1) unit in service utilization, and an increase of 1 unit in the knowledge of service results in an increase of 0.214 (=1.214 − 1) unit in service utilization. However, individual financial status and family care support are not significant predictors for the utilization of non-medical community healthcare services among the elderly.

Additionally, among control variables, education is significantly associated with the utilization of non-medical community healthcare services among the elderly across all models, and elderly having a higher level of education are more likely to use the services. Age is not associated with service utilization in Model 1 and Model 2. When demand-related factors are entered, age becomes positively associated with service utilization in Model 3 and Model 4. This suggests that there may be interaction between age and demand-related factors. Residency location is significantly associated with service utilization in Model 1 and Model 3. When supply-related factors are entered, residency location becomes no longer associated with service utilization in Model 2 and Model 4. This suggests that there may be interaction between residency location and supply-related factors. The other control variables are not associated with service utilization among the elderly.

## 4. Discussion

This study focused solely on non-medical community healthcare services, but not on medical community healthcare services. As noted in the findings, supply-related factors and demand-related factors are significantly associated with the utilization of non-medical community healthcare services among the elderly in China. This confirms what some scholars have suggested regarding the significant impacts of supply-related factors and demand-related factors in explaining healthcare service utilization [14,34]. Further discussion is as follows.

### 4.1. Supply-Related Factors

Similar to previous research [14,15], service supply is identified as a significant predictor for the utilization of non-medical community healthcare services among the elderly in this study, and more adequate supply of services predicts a higher level of service utilization. However, as noted in the findings, although the level of service supply is low, service supply exceeds service utilization. This indicates that there is waste of resources on the supply side, and there are other relevant factors effecting service utilization, apart from service supply. Furthermore, we find that service quality is also a predictor for service utilization, consistent with previous research [16]. As noted in the findings, the quality of non-medical community healthcare services is average, indicating that the elderly were not very satisfied with non-medical community healthcare services, as they were with medical community healthcare services [16]. This may make non-medical community healthcare services less attractive for the elderly and reduce service utilization. This may also be an important reason why service supply exceeds service utilization.

Service charge was usually considered a predictor for community healthcare service utilization in previous studies [8,17], especially for medical community healthcare service utilization [29]. However, in this study, service charge is not significantly associated with the utilization of non-medical community healthcare services among the elderly. It is well known that there is a common problem of expensive medical treatment in China, leading to service costs being an important factor affecting the utilization of medical community healthcare services among elderly Chinese [29]. Nevertheless, non-medical community healthcare services for the elderly have certain welfare characteristics, suggesting that the services are usually provided for the elderly at a low price, or even free of charge in China [6]. As noted in the findings, the charge for non-medical community healthcare services is generally low. Coupled with the great improvement in the individual financial status of elderly Chinese [5], for most of the elderly service charge may be no longer significant in predicting the utilization of non-medical community healthcare services.

In this study, service accessibility does not contribute in explaining the utilization of non-medical community healthcare services among the elderly. This finding is not consistent with previous studies in which service accessibility could explain the proportion of variations in service utilization [18,19]. On account of nonrandom sampling of the elderly, service accessibility may be good for all the respondents, but not good for old people living far away from community healthcare service centers, especially in the rural areas. In view of this, further research is highly recommended.

### 4.2. Demand-related Factors

Similar to previous studies [20], service need is also a predictor for the utilization of non-medical community healthcare services among the elderly in this study, and higher level of service need predicts a higher level of service utilization. However, as noted in the findings, although the level of service need is low, service need exceeds service utilization. This indicates that there are unmet needs on the demand side, and there are other relevant factors having an effect on service utilization, apart from service need. Furthermore, we find that knowledge of service is a significant predictor for service utilization, consistent with previous research [18,19]. In China, non-medical community healthcare services are still in their infancy, and they are something new for the elderly [5]. Lack of knowledge makes it virtually impossible for the users to make informed choices [44]. Having average knowledge about non-medical community healthcare services may impede service utilization among the elderly, even if they encounter service need. This may be an important reason why service need exceeds service utilization. Additionally, health status, primarily used to measure need factors in the Andersen model, was usually considered a significant predictor for medical community healthcare service utilization among the elderly [28,29]. However, in this study, the association of non-medical community healthcare service utilization is significant with perceived service need, but not with health status. This implies that there may be a big difference between the elderly’s needs for medical community healthcare services and for non-medical community healthcare services. In fact, individuals’ needs for medical healthcare services are mainly for treatment needs, and individuals’ needs for non-medical healthcare services are mainly for care needs [4]. This also suggests that it is reasonable to measure the need factors affecting non-medical community healthcare service utilization in terms of perceived service need instead of health status.

Individual financial status was usually considered a predictor for community healthcare service utilization in previous studies [11], especially for medical community healthcare service utilization [1,30]. However, in this study, individual financial status is not associated with the utilization of non-medical community healthcare services among the elderly. With the rapid development of the economy and the continuous improvement of the social security system, the individual financial status of elderly Chinese has been greatly improved [5]. In the context of generally low charges for non-medical community healthcare services, different from the expensive cost of medical community healthcare services, individual financial status is no longer a significant barrier for non-medical community healthcare service utilization among elderly Chinese. Even low-income elderly can use the services free of charge, supported by the government’s welfare policies [21].

Family care support was usually considered a predictor for community healthcare service utilization in previous studies [21], especially for medical community healthcare service utilization [45]. However, in this study, family care support is not associated with the utilization of non-medical community healthcare services among the elderly. In terms of medical community healthcare service utilization, family care support could reduce the barriers to service access and facilitate service utilization among the elderly [45]. Nevertheless, in terms of non-medical community healthcare service utilization, when the elderly are able to take care of themselves they are mainly served by themselves. When the elderly are not able to take care of themselves, all can basically receive care support from family members in their daily life. Since family care support for elderly Chinese is highly homogeneous, family care support is no longer associated with non-medical community healthcare service utilization among the elderly.

### 4.3. Limitations

This study has several limitations. Firstly, the size of the resulting sample may be somewhat small, and on account of nonrandom sampling of the elderly, the representativeness of the sample is not particularly good. The association of service utilization with service accessibility needs to be further examined. Secondly, since we used personal interviews and the respondents received a small incentive to participate in the survey, social desirability response bias might occur and additionally jeopardize the validity of the results. Thirdly, it has to be noted that the measurements of some variables were not particularly accurate, such as service supply, service need and service quality. The measurement of service quality is vague, since we used a proxy variable to represent an entire construct. The measurements of service supply and service need were designed by the authors, so the validity and reliability of these measures need to be further tested. Future research should develop better measurement tools for service supply and service need. Fourthly, for the purpose of this study, we primarily evaluated the main effects of supply and demand-related factors but did not evaluate the moderating effects of control variables. As noted in the findings, there may be moderating effects of age on the link between demand-related factors and service utilization, and residency location on the link between supply-related factors and service utilization. Further research efforts should take the moderating effects of these control variables into consideration. Finally, this study did not take healthcare outcomes into consideration. Since healthcare service utilization results in healthcare outcomes [35], further research is highly recommended.

## 5. Conclusions

Focusing solely on non-medical community healthcare services, we established an analytical framework for healthcare service utilization by reconstructing the Andersen behavioral model from the perspective of both the supply and demand sides. Based on this analytical framework, we examined supply and demand-related decisive factors in non-medical community healthcare service utilization among elderly Chinese using Poisson regression and the survey data from Shaanxi province, China. To our knowledge, this is the first study to examine both supply-related factors and demand-related factors affecting the utilization of non-medical community healthcare services among elderly Chinese. The main conclusions are as follows.

First, service supply and service quality from the supply side, and service need and knowledge of service from the demand side, are positively associated with the utilization of non-medical community healthcare services among elderly Chinese. Considering that there is waste of resources on the supply side and unmet needs on the demand side, to improve the utilization of non-medical community healthcare services among the elderly, we should consolidate the capacity of community healthcare service centers from the human, financial, material and other aspects in order to improve service quality [16], strengthen the publicity regarding non-medical community healthcare services through the media, free experience, door-to-door promotion and other ways in order to improve the elderly’s knowledge of the services [19], and provide services for the elderly based on their needs [38].

Second, other factors identified in previous studies, including service charge and service accessibility from the supply side and individual financial status and family care support from the demand side, are not significant predictors for the utilization of non-medical community healthcare services among the elderly in the context of China. Since non-medical community healthcare services for the elderly have certain welfare characteristics and the charge is generally low, the sustainability of non-medical community healthcare services should not be overlooked when community healthcare service centers cover more and more urban and rural communities. So, we should determine a reasonable price for the services to promote sustainable development [46].

Additionally, the Andersen behavioral model needs to be modified accordingly when applied to the study of non-medical community healthcare service utilization. It may be more reasonable to measure the need factors in the Andersen behavioral model in terms of perceived service need rather than health status. The analytical framework established in this study may extend the application of the Andersen behavioral model, and provide a new approach to explore the decisive factors in non-medical community healthcare service utilization. Nevertheless, considering that service supply and service need exceed service utilization, further studies, which shed light on additional reasons for underutilization of non-medical community healthcare services among the elderly in China, will need to be undertaken.

## Figures and Tables

**Table 1:** Measurements of variables.

Variable Type	Variable Name	Measurement Format	Response Options and Value Assignment	Source of Variable
Dependent variable	Service utilization	The number of service items used in the past six months	value = 0, 1, 2, 3, 4	Du & Wang [5]
Independent Variable: supply- related factors	Service supply	The number of service items offered by community	value =0, 1, 2, 3, 4	Kendig et al. [14]; Zheng & Kong [15]
	Service quality	Satisfaction with non-medical community healthcare services	1 = very dissatisfied, 2 = dissatisfied, 3 = average, 4 = satisfied, 5 = very satisfied	Gupta & Varsha [40]; Jandavath & Byram [41]
	Service charge	Price perception of non-medical community healthcare services	1 = very low, 2 = low, 3 = average, 4 = high, 5 = very high	Li & Wang [8]; Nelms et al. [17]
	Service accessibility	Convenience to community healthcare service center	1 = very inconvenient, 2 = inconvenient, 3 = average, 4 = convenient, 5 = very convenient	Di et al. [3]; Liu [18]; Chen & Chen [19]
Independent Variable: demand- related factors	Service need	The number of service items needed	value = 0, 1, 2, 3, 4	Crist et al. [20]
	Individual financial status	Personal annual income	Units of 10,000 RMB	Guo et al. [11]
	Family care support	Get care support from family members in daily life	0 = no, 1 = yes	Peng et al. [21]
	Knowledge of service	Knowing of non-medical community healthcare services	1 = very little, 2 = little, 3 = average, 4 = well, 5 = very well	Liu [18]; Chen & Chen [19]
Control variable	Age	Age in 2019	value ≥ 60	Fu et al. [1]
	Gender	Gender	0 = female, 1 = male	Shim et al. [23]
	Education	Level of education attained	1 = primary school and below, 2 = middle school, 3 = high school and above	Di et al. [3]
	Marital status	Marital status	0 = single, 1 = married	Lai [12]
	Health status	Self-perceived health	1 = very poor, 2 = poor, 3 = average, 4 = good, 5 = very good	Zhang& Tong [28]
	Residency location	Reside in the urban or rural areas	0 = rural, 1 = urban	Di et al. [3]

**Table 2:** Basic descriptive statistics of service utilization (N = 458).

Dependent Variable	Mean	Variance	Skewness
Service utilization	0.36	0.34	1.83

**Table 3:** Descriptive statistics of individual characteristics (N = 458).

Individual Characteristics	*n* (%)	Individual Characteristics	*n* (%)
Age		Marital status	
60–69	234 (51.09)	married	333 (72.71)
70–79	166 (36.24)	single	125 (27.29)
80–89	57 (12.45)	Health status	
Above 90	1 (0.22)	Very poor	15 (3.28)
Gender		poor	81 (17.69)
Male	178 (38.86)	Average	128 (27.95)
Female	280 (61.14)	good	158 (34.5)
Education		Very good	76 (16.59)
primary school and below	224 (48.91)	Residency location	
middle school	124 (27.07)	urban	190 (41.48)
high school and above	110 (24.02)	rural	268 (58.52)

**Table 4:** Descriptive statistics of dependent variable and independent variables.

Variable Type	Variable Name	Mean (SD)	*n* (%)
Dependent variable	Service utilization (N = 458)	0.36 (0.58)	
	0 item		310 (67.69)
	1 item		134 (29.26)
	2 item		10 (2.18)
	3 item		3 (0.66)
	4 item		1 (0.22)
Independent Variable: supply-related factors	Service supply (N = 458)	1.64 (1.20)	
	0 item		73 (15.94)
	1 item		175 (38.21)
	2 item		102 (22.27)
	3 item		61 (13.32)
	4 item		47 (10.26)
	Service quality (N = 385)	3.27 (1.06)	
	Service charge (N = 385)	1.47 (0.87)	
	Service accessibility (N = 458)	4.21 (1.02)	
Independent Variable: Demand -related factors	Service need (N = 458)	1.51 (1.60)	
	0 item		204 (44.54)
	1 item		54 (11.79)
	2 item		52 (11.35)
	3 item		59 (12.88)
	4 item		89 (19.43)
	Individual financial status (N = 458)	2.13 (2.56) ^†^	
	Family care support (Yes) (N = 458)		380 (82.53)
	Knowledge of service (N = 458)	3.32 (1.25)	

^†^ Unit of measurement is ten thousand (10,000) RMB.

**Table 5:** Poisson regression analysis of associations between supply and demand-related factors and service utilization (N = 458).

Parameter		Model 1	Model 2	Model 3	Model 4
		B (Exp(B))	B (Exp(B))	B (Exp(B))	B (Exp(B))
Control variable	Age	0.020 (1.021)	0.023 (1.023)	0.025 (1.026) *	0.026 (1.027) *
	Gender (Male)	−0.121 (0.886)	−0.143 (0.867)	−0.137 (0.876)	−0.128 (0.880)
	Education				
	middle school	−0.472 (0.624) *	−0.505 (0.603) *	−0.450 (0.637) *	−0.496 (0.609) *
	primary school and below	−0.696 (0.499) ***	−0.622 (0.537) **	−0.621 (0.537) **	−0.548 (0.578) *
	Marital status(married)	−0.357 (0.700) *	−0.232 (0.793)	−0.116 (0.890)	−0.073 (0.930)
	Health status	−0.018 (0.983)	−0.037 (0.964)	−0.027 (0.973)	−0.050 (0.951)
	Residency location (urban)	0.516 (1.676) **	0.273 (1.314)	0.512 (1.669) **	0.301 (1.351)
Supply- related factors	Service supply		0.298 (1.347) ***		0.282 (1.325) ***
	Service quality		0.177 (1.194) *		0.131 (1.140) *
	Service charge		−0.023 (0.977)		−0.064 (0.938)
	Service accessibility		−0.023 (0.977)		−0.050 (0.952)
Demand- related factors	Service need			0.064 (1.066) *	0.084 (1.088) *
	Individual financial status			−0.008 (1.000)	−0.006 (1.000)
	Family care support (Yes)			−0.200 (0.818)	−0.116 (0.891)
	Knowledge of service			0.256(1.292) ***	0.194 (1.214) **
−2Log likelihood		40.223 ***	69.553 ***	58.459 ***	81.190 ***
Change of −2Log likelihood			58.660 ***	18.236 ***	40.966 ***

* *p* < 0.05, ** *p* < 0.01, *** *p* < 0.001.

## Data Availability

The data presented in this study are available on request from the corresponding author. The data are not publicly available due to privacy.

## References

[1] Fu X., Sun N., Xu F., Li F., Sun C. (2018). Influencing factors of inequity in health services utilization among the elderly in China. Int. J. Equity Health.

[2] Xie G. (2020). Thinking about work injury of retirees. Chin. Soc. Sec..

[3] Di X., Wang L., Dai X., Yang L. (2020). Assessing the accessibility of home-based healthcare services for the elderly: A case from Shaanxi province, China. Int. J. Environ. Res. Public Health.

[4] Cox L.E., Brennan M., Brennan M., DeMarco R. (2017). Medical, social and supportive services for older adults with HIV. HIV and Aging.

[5] Du P., Wang Y. (2017). Determinants of utilization of social care service for older persons in China. Pop. Res..

[6] Wang Z. (2018). Policy Analysis and governance model reconstruction of the provision of home-based community care services. Probe.

[7] Dai L. (2018). Combination of medical and non-medical care: A Chinese program for healthy aging. Chin. Med. Her..

[8] Li F., Wang Y. (2016). Current situation of the utilization of the community elderly care service and its influencing factors. Pop. Soc..

[9] Jiang Z. (2020). Steadily advance the strategy of a healthy China. Chin. Dev. Obs..

[10] Sarah H., Guido F., Joanna G., Johan V., Maurits V., Guido V. (2012). Health-care and home-care utilization among frail elderly persons in Belgium. Eur. J. Public Health.

[11] Guo C., Du W., Hu C., Zheng X. (2015). Prevalence and factors associated with healthcare service use among Chinese elderly with disabilities. J. Public Health.

[12] Lai D. (2004). Use of home care services by elderly Chinese immigrants. Home Health Care Serv. Q..

[13] Murphy C.M., Whelan B.J., Normand C. (2015). Formal home-care utilisation by older adults in Ireland: Evidence from the Irish Longitudinal Study on Ageing. Health Soc. Care Community.

[14] Kendig H., Mealing N., Carr R., Lujic S., Byles J., Jorm L. (2012). Assessing patterns of home and community care service use and client profiles in Australia: A cluster analysis approach using linked data. Health Soc. Care Community.

[15] Zheng L., Kong L. (2017). Factors influencing the utilization of home care services for the elderly and differences between urban and rural areas. St. Dec..

[16] Ding Z., Qu J. (2019). Research on equalization of community home care services in China. Pop. J..

[17] Nelms L., Johnson V., Teshuva K., Foreman P., Stanley J. (2009). Social and health factors affecting community service use by vulnerable older people. Aust. Soc. Work.

[18] Liu Y. (2003). Aging service need and use among chinese american seniors: Intragroup variations. J. Cross Cult. Gerontol..

[19] Chen Y., Chen H. (2017). Disparity between need and use: Reflections on delivery of community home care services. Zhejiang. Acad. J..

[20] Crist J.D., Seon H.W., Choi M. (2007). A comparison of the use of home care services by Anglo-American and Mexican American elders. J. Transcult. Nurs..

[21] Peng X., Song L., Huang J. (2017). Determinants of long-term care services among disabled older adults in China. Pop. Res..

[22] Burns M.J., Cain V.A., Husaini B.A. (2001). Depression, service utilization, and treatment costs among medicare elderly: Gender differences. Home Health Care Serv. Q..

[23] Shim H., Ailshire J., Crimmins E. (2018). The Health and Retirement Study: Analysis of associations between use of the internet for health information and use of health services at multiple time points. J. Med. Internet Res..

[24] Liu L. (2014). The health heterogeneity of and health care utilization by the elderly in Taiwan. Int. J. Environ. Res. Public Health.

[25] Kim E., Kim K., Yoo D. (2016). Comparison of medical costs, health care utilization, and health outcomes between the elderly with and without disabilities in Korea. Value Health.

[26] Ensrud K.E., Kats A.M., Schousboe J.T., Taylor B.C., Vo T.N., Cawthon P.M., Hoffman A.R., Langsetmo L. (2018). Frailty phenotype and healthcare costs and utilization in older women. J. Am. Geriatr. Soc..

[27] Lartey S., Graaff B., Magnussen C. (2020). Health service utilization and direct healthcare costs associated with obesity in older adult population in Ghana. Health Policy Plan..

[28] Zhang L., Tong X. (2014). Utilization of in-patient service and their influencing factors among the elderly in rural China. Nanjing. J. Soc. Sci..

[29] Hu J., Liu Y., Huang C. (2017). Potential medical need of the poor elderly in rural China. Econ. Rev..

[30] Kwon I., Shin O., Park S., Kwon G. (2019). Multi-morbid health profiles and specialty healthcare service use: A moderating role of poverty. Int. J. Environ. Res. Public Health.

[31] Macinko J., Andrade F.B.D., Souza Junior P.R.B.D., Lima-Costa M.F. (2018). Primary care and healthcare utilization among older Brazilians. Rev. Saude Publica.

[32] Hu X., Zhang H., Sun X., Gu Y., Dong H. (2019). Older adults’ choices of first-contact care and related factors in Zhejiang and Qinghai Province, China. Geriatr. Gerontol. Int..

[33] Ishizuki T., Wake J. (2010). Factors associated with home care service use among severely disabled elderly: A comparison between long-term community dwellers and nursing home residents. Jpn. J. Soc. Welf..

[34] Babitsch B., Gohl D., Lengerke T. (2012). Re-revisiting Andersen’s behavioral model of health services use: A systematic review of studies from 1998–2011. GMS Psycho-Soc. Med..

[35] Andersen R.M., Davidson P.L., Baumeister S.E., Kominski E.F. (2013). Improving access to care in America. Changing the U.S. Health Care System: Key Issues in Health Services, Policy, and Management.

[36] Lengerke T., Gohl D., Babitsch B., Swart C., Lengerke T. (2014). Re-revisiting the behavioral model of health care utilization by Andersen: A review on theoretical advances and perspectives. Health Care Utilization in Germany.

[37] Lei X. (2019). Study on the satisfaction of medical services between urban and rural residents. J. Agrotech. Econ..

[38] Zhang H., Luo W. (2019). A study of the influence of care service resources on the demand for social care service for senior citizens. Chin. Pop. Res. Environ..

[39] Upadhyai R., Jain A., Roy H., Pant V. (2019). A Review of Healthcare Service Quality Dimensions and their Measurement. J. Health Man..

[40] Gupta K., Varsha R. (2016). Importance of quality in health care sector: A review. J. Health Man..

[41] Jandavath R., Byram A. (2016). Healthcare service quality effect on patient satisfaction and behavioural intentions in corporate hospitals in India. Int. J. Pharm. Healthc. Mark..

[42] Calvin J.A. (1998). Regression models for categorical and limited dependent variables. Technometrics.

[43] Du P., Sun J., Zhang W., Wang X. (2016). The demands of old-age care and the family and social resources for the Chinese elderly. Pop. Res..

[44] Moberg L., Blomqvist P., Winblad U. (2016). User choice in Swedish eldercare-conditions for informed choice and enhanced service quality. J. Eur. Soc. Policy.

[45] Liao X., Luo J., Luo Y. (2017). The effect of intergenerational support on medical service utilization among rural elderly. Pop. Dev..

[46] Lin B. (2017). “Low-level equilibrium trap” and policy supports in elderly services. J. Xinjiang. Norm. Univ..

